# Expressions of COX-2 and VEGF-C in gastric cancer: correlations with lymphangiogenesis and prognostic implications

**DOI:** 10.1186/1756-9966-30-14

**Published:** 2011-01-28

**Authors:** Hong-Feng Gou, Xin-Chuan Chen, Jiang Zhu, Ming Jiang, Yu Yang, Dan Cao, Mei Hou

**Affiliations:** 1 Center of Medical Oncology, West China Hospital, Sichuan University, PR China; 2 Department of hematology, West China Hospital, Sichuan University, PR China

## Abstract

**Background:**

Cyclooxygenase-2 (COX-2) has recently been considered to promote lymphangiogenesis by up-regulating vascular endothelial growth factor-C (VEGF-C) in breast and lung cancer. However, the impact of COX-2 on lymphangiogenesis of gastric cancer remains unclear. This study aims to test the expression of COX-2 and VEGF-C in human gastric cancer, and to analyze the correlation with lymphatic vessel density (LVD), clinicopathologic features and survival prognosis.

**Methods:**

Using immunohistochemistry, COX-2, VEGF-C and level of LVD were analyzed in 56 R0-resected primary gastric adenocarcinomas, while paracancerous normal mucosal tissues were also collected as control from 25 concurrent patients. The relationships among COX-2 and VEGF-C expression, LVD, and clinicopathologic parameters were analyzed. The correlations of COX-2, VEGF-C and level of LVD with patient prognosis were also evaluated by univariate tests and multivariate Cox regression.

**Results:**

The expression rates of COX-2 and VEGF-C were 69.64% and 55.36%, respectively, in gastric carcinoma. Peritumoral LVD was significantly higher than that in both normal and intratumoral tissue (*P *< 0.05). It was significantly correlated with lymph node metastasis and invasion depth (*P *= 0.003, *P *= 0.05). VEGF-C was significantly associated with peritumoral LVD (*r *= 0.308, *P *= 0.021). However, COX-2 was not correlated with VEGF-C (*r *= 0.110, *P *= 0.419) or LVD (*r *= 0.042, *P *= 0.758). Univariate analysis showed that survival time was impaired by higher COX-2 expression and higher peritumoral LVD. Multivariate survival analysis showed that age, COX-2 expression and peritumoral LVD were independent prognostic factors.

**Conclusions:**

Although COX-2 expression was associated with survival time, it was not correlated with VEGF-C and peritumoral LVD. Our data did not show that overexpression of COX-2 promotes tumor lymphangiogenesis through an up-regulation of VEGF-C expression in gastric carcinoma. Age, COX-2 and peritumoral LVD were independent prognostic factors for human gastric carcinoma.

## Background

Gastric carcinoma is one of the most common digestive malignancies in the world, especially in East and Southeast Asia, including China [1]. Regional lymph nodes are the most common site of metastasis while lymph node metastasis is a major prognostic factor in gastric carcinomas. Understanding the mechanisms of lymphatic metastasis represents a crucial step and may result in a new therapeutic target in the treatment of human cancer. Lymphatic metastasis was previously believed to occur through pre-existing lymphatics [2,3]. However, recent studies have suggested that lymphangiogenesis, the formation of new lymphatic vessels induced by tumors, is directly correlated with the extent of lymph node metastasis of solid tumors [4,5]. The degree of lymphatic vessel density (LVD) can quantify tumor lymphangiogenesis.

LVD of cancer tissue has been considered one of the prognostic factors for survival outcome in various cancers including gastric carcinoma [6,7]. Vascular endothelial growth factor-C (VEGF-C) is the most important lymphangiogenic factor produced by tumor and stromal cells. It has been found that VEGF-C is strongly expressed and has become an important predictor of lymphangiogenesis and prognosis in numerous types of cancers, including gastric carcinoma [8-10]. VEGF-C can promote lymphangiogenesis and lymph node metastasis of tumors by activating its special receptor vascular endothelial growth factor receptor-3 (VEGFR-3) [11,12].

Cyclooxygenase-2 (COX-2) is the rate-limiting enzyme in prostaglandin synthesis and has been reported to be overexpressed in various human cancers. During the progression of a cancer, COX-2 takes part in many pathophysiologic processes, including cell proliferation, apoptosis, modulation of the immune system, and angiogenesis [13-17]. The role of COX-2 in angiogenesis of human cancers is well-documented and VEGF-A was identified as a major downstream effector gene of COX-2-induced angiogenesis in human cancer [18,19]. In contrast to the effect of COX-2 on angiogenesis, the effects on lymphangiogenesis and lymphatic metastasis remain poorly understood. Recently, some studies have found that COX-2 expression is highly correlated with lymph node metastasis [20,21]. Several lines of experimental evidence have shown that COX-2 might stimulate VEGFR-3 to promote lymphangiogenesis by up-regulating VEGF-C in breast and lung cancer cells [22,23].

However, the role of COX-2 in lymphangiogenesis of gastric carcinoma remains unclear. Using immunohistochemistry, our study aimed to detect the expression of COX-2 and VEGF-C protein and the levels of lymphatic vessel density (LVD) in human gastric cancer and analyze their correlations with clinicopathological characteristics and prognosis.

## Methods

### Patients and specimens

Fifty-six patients with histologically proven gastric adenocarcinoma and who underwent radical gastrectomy at West China Hospital, Sichuan University, China between January 2001 and October 2002, were included in the present investigation. In this investigation, paracancerous normal mucosal tissues from 25 patients were collected as a control. Patients undergoing neoadjuvant chemotherapy and/or radiotherapy were excluded. TNM staging was carried out according to the American Joint Committee on Cancer (AJCC) classification, and historical grading was performed according to WHO criteria. Paraffin-embedded, formalin-fixed surgical specimens were prepared and collected for immunohistochemical staining.

### Immunohistochemical staining

Specimens were immunostained with the standard labeled streptavidin-biotin protocol. Briefly, after deparaffinization and antigen retrieval, 4-μm tissue sections were incubated with COX-2 antibodies (monoclonal rabbit anti-human, 1:100, Goldenbridge Biotechnology Co, Ltd, Beijing, China) and VEGF-C antibodies (polyclonal rabbit anti-human, 1:100, Goldenbridge Biotechnology Co., Ltd) at 37°C for 1 h then at 4°C overnight. The sections were then incubated with biotinylated goat anti-rabbit immunoglobulin G (1:200, Zymed Laboratories Inc, USA) and subsequently incubated with horseradish labeled streptavidin (1:200, Zymed Laboratories Inc). 3,3'-Diaminobenzidine was used as a chromogen and hematoxylin as a counterstain. For the staining of lymphatic vessels, a rabbit anti-human D2-40 polyclonal antibody (rabbit polyclonal, Dako Denmark A/S Co., Denmark) was used. The procedure for immunohistochemical staining of D2-40 is similar to that of the COX-2 staining at a dilution of 1:100.

### Evaluation of immunohistochemical staining

The immunohistochemical score (IHS) based on the German immunoreactive score was used for COX-2 and VEGF-C immunohistochemical evaluation [24]. The IHS is calculated by combining the quantity score (percentage of positive stained cells) with the staining intensity score. The quantity score ranges from 0 to 4, i.e. 0, no immunostaining; 1, 1-10% of cells are stained; 2, 11-50% are positive; 3, 51-80% are positive; and 4, ≥81% of cells are positive. The staining intensity was scored as: 0 (negative), 1 (weak), 2 (moderate) and 3 (strong). Raw data were converted to IHS by multiplying the quantity score (0-4) by the staining intensity score (0-3). Theoretically, the scores can range from 0 to 12. An IHS of 9-12 was considered a strong immunoreactivity; 5-8, moderate; 1-4, weak; and 0, negative. In statistical analysis, COX-2 and VEGF-C scores were placed in a high expression group (strong and moderate immunoreactivity) and a low expression group (weak and negative immunoreactivity). Immunoreactivity was scored by two independent researchers.

LVD was detected by immunostaining for D2-40, according to the criteria of Masakau *et al. *[25]. First, areas with highly D2-40-positive vessels (hot spots) in peritumoral, intratumoral and normal tissue were identified, by scanning the sections at low magnification (×100); then the number of D2-40 positive vessels was counted in five high-magnification fields (×400) for each case. The mean value for the five fields was calculated as the LVD for each tumor. To evaluate the impact of LVD on prognosis, we divided the 56 cases into two groups according to the mean LVD level.

### Statistical analysis

Statistical analyses were performed with SPSS 11.5 software (SPSS Inc, Chicago, USA). The correlations among the expression of COX-2, VEGF-C, levels of LVD, and clinicopathologic characteristics were calculated by Student's *t*-test, chi-square correlation test and Spearman's coefficient of correlation as appropriate. The Kaplan-Meier method was used to estimate survival as a function of time, and survival differences were analyzed with the log-rank test. A multivariable test was performed to determine the factor correlated with survival length by Cox regression analysis. The statistical significance level was defined as *P *< 0.05.

## Results

### Patient information

The 56 patients (35 males and 21 females) had a mean age of 56.2 (range 27-74) years. Twenty-six of the cases displayed weight loss, and 17 presented anemia with hemoglobin (HGB) < 90 g/l. Histological examination showed that 4 displayed well differentiated adenocarcinoma, 18 moderate and 34 poor. According to the sixth AJCC TNM classification, 16 patients were in stage I, 18 in stage II, 19 in stage III, and 3 in stage IV. Of the 56 patients, 39 (69.6%) had lymph node metastasis. Up to 2008, there were 32 patients in total that had died.

### COX-2, VEGF-C and D2-40 expression in gastric carcinoma

**Figure 1:**
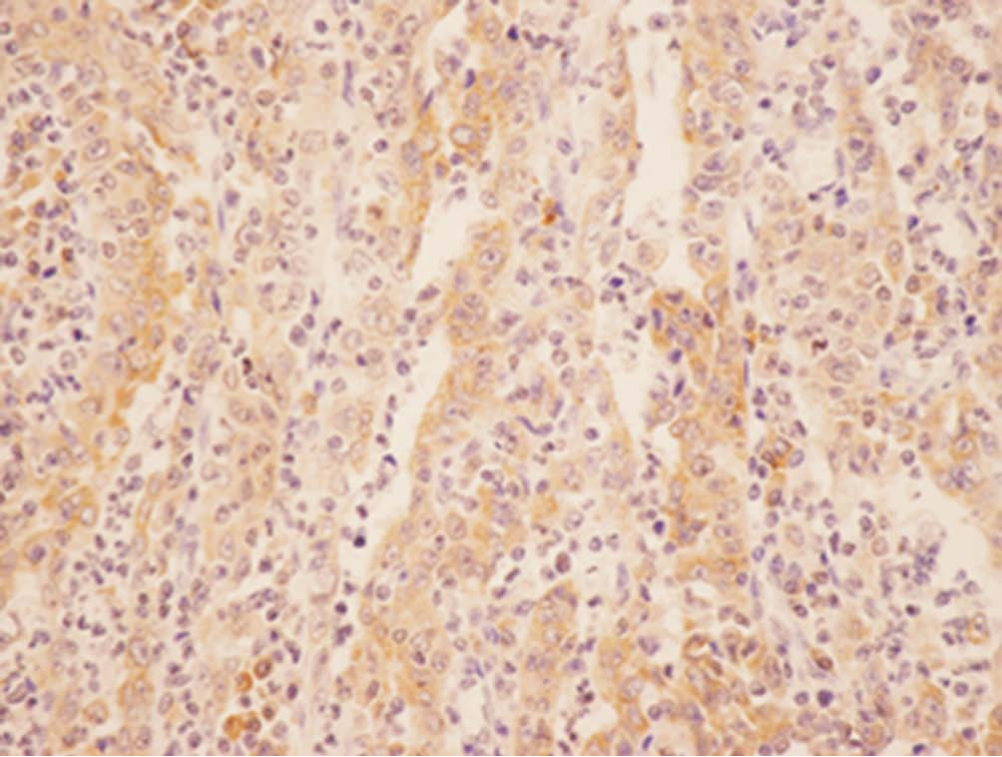
**Immunohistochemical staining of Cox-2 in the gastric carcinoma: the positive expression of COX-2 protein was stained as yellow or brownish yellow in the cytoplasm of carcinoma cells (LsAB, ×400)**.

**Figure 2:**
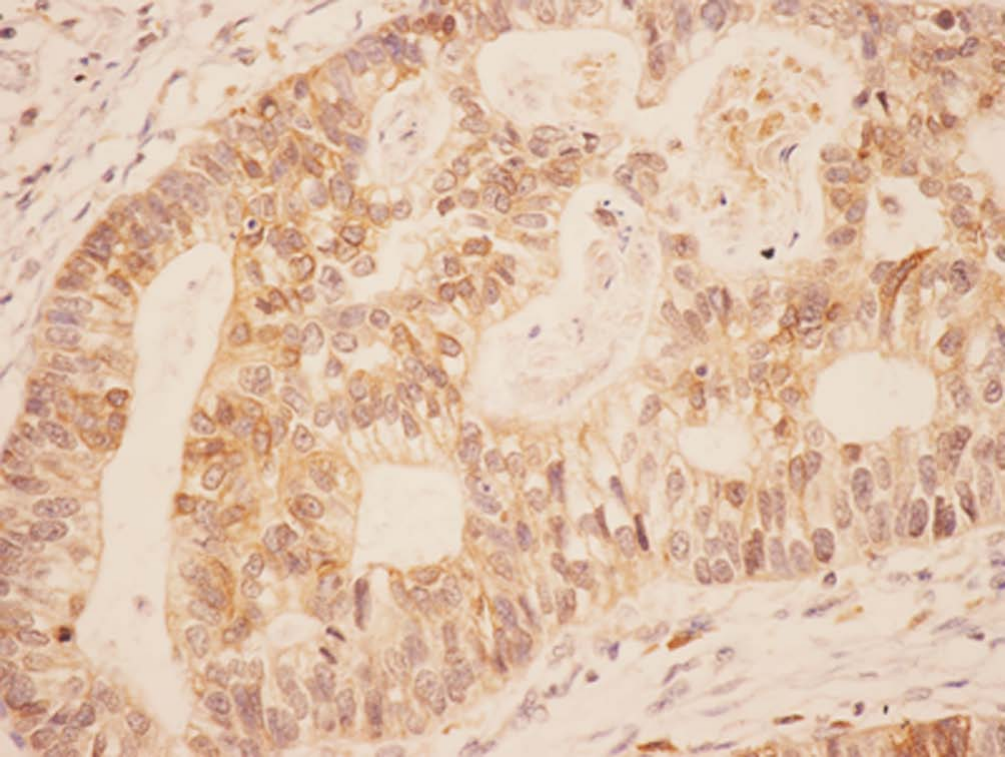
Immunohistochemical staining of VEGF-C in the gastric carcinoma: the positive expression of VEGF-C protein was stained as yellow or brownish yellow in the cytoplasm of carcinoma cells (LsAB, ×400).

Positive expression of COX-2 protein and VEGF-C showed as a yellow or brownish yellow stain in the cytoplasm of carcinoma cells (Figures 1 and 2). The expression rates of COX-2 and VEGF-C were 69.64% (39/56) and 55.36% (31/56), respectively, in gastric carcinoma. However, normal tissue showed no immunoreactivity for COX-2 and VEGF-C.

**Figure 3:**
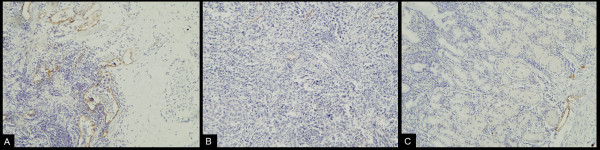
**Immunohistochemical staining of D2-40: Immunoreactivity of D2-40 proteins was found in the cytoplasm and cellular membrane of lymphatic endothelial cells.** A. Detection of lymphatic vessels in the peritumoral tissue of gastric carcinoma was highlighted by immunostaining against D2-40 (LsAB,×200). B. Immunohistochemical staining of D2-40 in the intratumoral tissue of gastric carcinoma (LsAB, ×200). C. Immunohistochemical staining of D2-40 the normal gastric mucosal tissue (LsAB, ×200).

Immunoreactivity of D2-40 proteins was found in the cytoplasm and cellular membrane of lymphatic endothelial cells. The distribution of D2-40-positive cells was frequently located in peritumoral tissue (hot spot) (Figure 3A). The means of LVD in peritumoral, intratumoral and normal tissue of the 56 gastric carcinomas were 9.24 ± 4.51, 2.88 ± 2.04, 2.69 ± 1.78, respectively. The LVD in peritumoral, intratumoral (Figure 3B) and normal tissue (Figure 3C) was significantly different by variance analysis of randomized block design. When compared to each other by least significant difference (LSD) test, there was a significant difference between the peritumoral LVD and both the intratumoural LVD and the LVD of normal tissue. There was no significant difference between the intratumoral LVD and the LVD of normal tissue. When the mean peritumoral LVD of 9.24 was chosen as the cut-off point for discrimination of the 56 patients, 32 patients were categorized in the low LVD group and 24 in the high LVD group.

### Correlation between COX-2, VEGF-C and LVD and clinicopathologic characteristics

The correlation of COX-2, VEGF-C and peritumoral LVD with clinicopathologic factors in gastric carcinoma is shown in Table 1. There was no significant correlation between COX-2 expression and any clinicopathologic characteristics, including gender, age, lymph node metastasis, histological differentiation, invasion depth and TNM stage (*P *> 0.05, chi-square test). Similarly, VEGF-C expression was not correlated with any clinicopathologic characteristics (*P *> 0.05, chi-square test). The peritumoral LVD was significantly correlated with lymph node metastasis and invasion depth. It was higher in the lymph node metastasis group (10.37 ± 4.61) than in the no lymph node metastasis group (6.64 ± 3.01) (*P *= 0.003, *t*-test) and was higher in the T3,T4 group (10.80 ± 5.24) than in the T1,T2 group (8.37 ± 3.85) (*P *= 0.05, *t*-test). No significant correlation was observed with the rest of the clinicopathologic parameters (*P *> 0.05, *t*-test).

**Table 1:** Correlation between COX-2, VEGF-C, peritumoral LVD and clinicopathologic factors in gastric carcinoma

Parameters	N	COX-2 expression			VEGF-C expression			LVD	
		Low	High	P value	Low	High	P value	Mean ± SD	P value
Histological grading									
Low	34	11	23	0.916	16	18	0.703	9.03 ± 4.37	0.721
Moderate	18	5	13		8	10		9.88 ± 5.15	
Well	4	1	3		1	3		8.14 ± 2.69	
Depth of invasion									
T1+T2	36	12	24	0.516	17	19	0.602	8.37 ± 3.85	0.052
T3+T4	20	5	15		8	12		10.80 ± 5.24	
Lymph node metastasis									
No	17	5	12	0.919	10	7	0.159	6.64 ± 3.01	0.003
Yes	39	12	27		15	24		10.37 ± 4.61	
TNM stage									
I+ II	34	11	23	0.686	18	16	0.12	8.40 ± 3.95	0.084
III+IV	22	6	16		7	15		10.53 ± 5.08	

### Correlation between COX-2, VEGF-C and LVD

The expression of COX-2 was not significantly correlated with VEGF-C expression (*r *= 0.110, *P *> 0.419) and peritumoral LVD (*r *= 0.042, *P *> 0.05). Peritumoral LVD in VEGF-C positive expression gastric carcinoma was 10.45 ± 5.11, which was significantly higher than that in VEGF-C negative expression gastric carcinoma (7.73 ± 3.09, *P *= 0.023). Peritumoral LVD was significantly associated with VEGF-C (*r *= 0.308, *P *= 0.021) (Table 2).

**Table 2:** Correlation between COX-2 and VEGF-C, peritumoral LVD

		COX-2	peritumoral LVD
VEGF-C	Coefficient	0.110	0.308
	P value	0.419	0.021
COX-2	Coefficient		0.042
	P value		0.758

### Survival analyses

#### Univariate prognostic analyses

**Figure 4:**
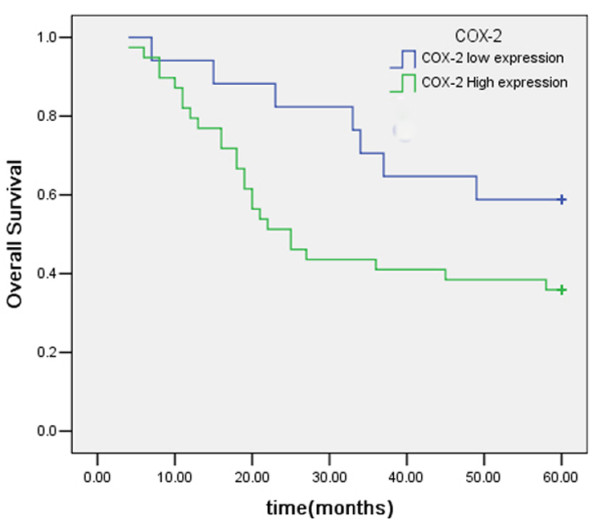
Kaplan-Meier overall survival curves for 56 patients with gastric carcinoma patients with COX-2 positive expression had a significantly worse OS compared with those with COX-2 negative expression.

**Figure 5:**
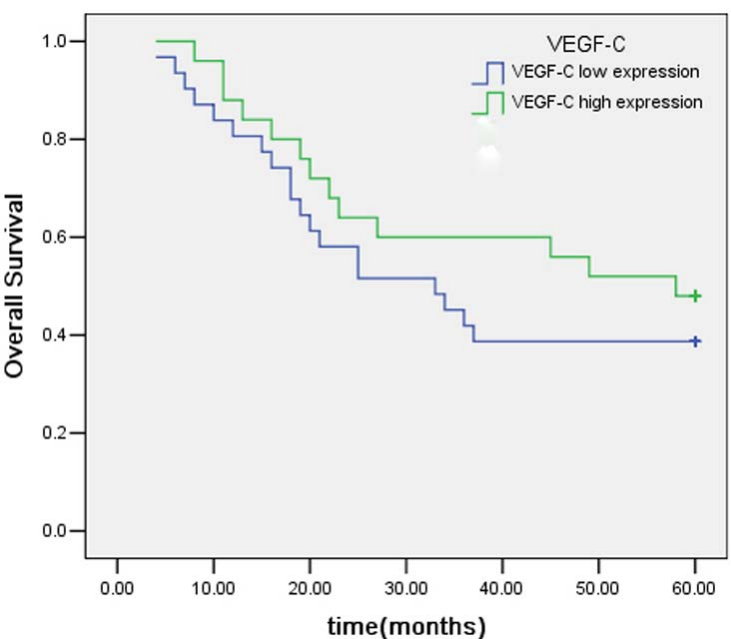
Kaplan-Meier overall survival curves for 56 patients with gastric carcinoma: patients with VEGF-C expression had no association with survival time of gastric carcinoma.

**Figure 6:**
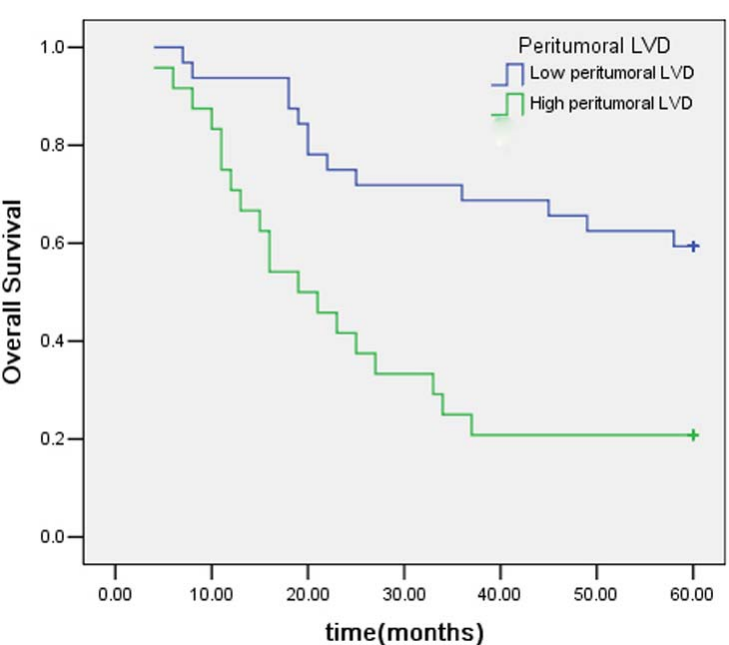
Kaplan-Meier overall survival curves for 56 patients with gastric carcinoma: patients with high peritumoral LVD had a significantly worse OS compared with those with low peritumoral LVD.

Within a total follow-up period of 60 months, 32 of the 56 assessable cases had died. The 5-year overall survival (OS) for all patients was 42.9%. Analysis of the impact of COX-2 status is shown in Figure 4. Six cases had died in the COX-2 low expression group and the 5-year OS was 64.7% whereas 26 cases had died in the COX-2 high expression group and the 5-year OS was 33.3%. Patients with high COX-2 expression tended to have poorer prognosis than patients with low COX-2 expression (*P *= 0.026, log-rank test). The 5-year OS of patients with low and high VEGF-C expression was 48% and 38.71%, respectively. Kaplan-Meier curves of overall survival stratified by VEGF-C status are shown in Figure 5. The survival time of patients in different expression groups showed no significant difference (*P *> 0.05, log-rank test). Analysis of the impact of LVD status is shown in Figure 6. The 5-year OS of patients with low and high LVD was 59.4% and 20.8%, respectively. Patients with high peritumoral LVD tended to have poorer prognosis than patients with low peritumoral LVD (*P *= 0.001, log-rank test).

#### Multivariate analysis and Cox's proportional hazard model

In Cox regression for OS including patients' age, gender, lymph node metastasis, histological differentiation, invasion depth, stage, COX-2 expression, VEGF-C expression, and peritumoral LVD, only age (*P *= 0.015, RR = 2.891, 95% confidence interval, 1.228-6.805), COX-2 expression (*P *= 0.021, RR = 3.244, 95% confidence interval, 1.192-8.828) and peritumoral LVD (*P *= 0.001, RR = 4.292, 95% confidence interval, 1.778-10.360) remained as independent prognostic factors.

## Discussion

The occurrence of lymphangiogenesis can be detected using several lymphatic vessel-specific markers. Previously, the lack of specific lymphatic molecular markers for lymphatic endothelium was the main obstacle to studying tumor lymphangiogenesis. D2-40, a novel monoclonal antibody, is a selective marker of lymphatic endothelium. It is specifically expressed on lymphatic but not vascular endothelial cells, compared with traditional lymphatic endothelium markers [26-28]. In this study, as shown in the results, D2-40 is only expressed in lymphatics and is negative in blood vessels and the distribution of D2-40 positive cells is exclusively in peritumoral tissue.

In the present study, the LVD of peritumoral tissue was significantly higher than that in both normal and intratumoral tissue. Peritumoral LVD is significantly related to the depth of invasion, lymph node metastasis and prognosis. Patients with high peritumoral LVD tend to have a poorer prognosis than patients with low peritumoral LVD. The role of intratumoral versus peritumoral lymphatics for lymph node metastasis remains controversial. Many studies have found an increased LVD in peritumoral tissue and peritumoral lymphangiogenesis is significantly correlated with lymph node metastasis and prognosis in human solid cancer [2,29-33]. However, the presence or absence of intratumoral lymphangiogenesis and the functional significance of intratumoral lymphatic vessels remain controversial [3]. Several studies have found lymphatics only in peritumoral tissue [34,35]. Padera *et al. *have reported that tumor cells are not able to metastasis by intratumoral lymphatic vessels [2], but other studies have demonstrated that the presence of intratumoral lymphangiogenesis and intratumoral LVD are correlated with lymph node metastasis and prognosis in several tumors [36-38].

Among the reported transduction systems in lymphangiogenesis in humans, the VEGF-C/VEGFR-3 axis is the main system [12,39]. VEGF-C is vital for the lymphangiogenic process supported by transgenic and gene deletion animal models [40-42]. It has been shown to be expressed highly and has a negative influence on prognosis and a positive correlation with lymph node metastasis including gastric carcinoma [8-10,43,44]. However, Arinaga *et al. *found that there was no significant correlation between VEGF-C and lymph node metastasis in non-small cell lung carcinoma [45]. In a univariate analysis, Möbius *et al. *reported that tumoral VEGF-C expression of adenocarcinoma of the esophagus was not a significant prognostic factor [46]. Our results showed that primary gastric carcinoma tissue elevated the expression of VEGF-C. However, there was no significant association between the expression rate of VEGF-C and clinicopathologic parameters. Probably, these discrepancies were influenced by intratumoral heterogeneity and the population size. But, in this study, there was a positive correlation between the expression of VEGF-C and peritumoral LVD.

The overexpression of COX-2 has been detected in several types of human cancer including colon, lung, stomach, pancreas and breast cancer and is usually associated with poor prognostic outcome. Cox-2 mRNA and protein were first found to be expressed in human gastric carcinoma by Ristimaki *et al. *in 1997 [47]. Previous studies show conflicting prognostic significance of COX-2 in gastric carcinoma. Johanna *et al. *found that there was a significant association between COX-2 expression and lymph node metastasis and invasive depth, and high COX-2 is an independent prognostic factor in gastric cancer [48]. However, contrary to the above results, some studies have shown that there was no association between COX-2 expression and prognosis [49]. Lim also found that there was no correlation between clinicopathological characteristics of gastric cancer patients and intensity of COX-2 protein expression [50]. In our study, we also found that COX-2 protein was expressed in cases of gastric carcinoma, but we did not find a significant association between COX-2 expression and clinicopathological characteristics. In this study, from univariate and multivariate analyses, we found a significant association between COX-2 expression and a reduced survival of patients with gastric cancer. These discrepancies are likely influenced by differences in study size, COX-2 detection methods, and criteria for COX-2 overexpression. These findings warrant larger studies with multivariate analysis to clarify the association of COX-2 with clinicopathological characteristics and poor prognosis in patients with gastric cancer.

In contrast to the effect of COX-2 on angiogenesis, the effect on lymphangiogenesis and lymphatic metastasis remains poorly understood. Recent studies suggest that COX-2 may play a role in tumor lymphangiogenesis through an up-regulation of VEGF-C expression. VEGF-C is the most important lymphangiogenic factor produced by tumor and stromal cells. Su *et al. *[23] found that lung adenocarcinoma cell lines transfected with Cox-2 gene or exposed to prostaglandin E2 caused a significant elevation of VEGF-C mRNA and protein. The authors suggested that Cox-2 up-regulated VEGF-C by an EP1 prostaglandin receptor and human epidermal growth factor receptor HER-2/Neu-dependent pathway. In addition, immunohistochemical staining of 59 lung adenocarcinoma specimens reflected a close association between COX-2 and VEGF-C. Kyzas *et al. *[51] found that there was a significant correlation between COX-2 expression and VEGF-C expression, and lymph node metastasis in head and neck cancer. Timoshenko *et al. *[22] found that VEGF-C expression and secretion could be inhibited by down-regulation of COX-2 with COX-2 siRNA in human breast cancer. Several reports have also revealed that there was a significant association between COX-2 expression and lymph node metastasis, and COX-2 expression was correlated with VEGF-C expression in gastric carcinoma [20,52]. These results indicated that a lymphangiogenic pathway, in which COX-2 up-regulated VEGF-C expression, might exist in human carcinoma. However, contrary to the above results, some studies have shown that there was no association between COX-2 expression and lymph node metastasis in many types of cancer, including gastric carcinoma [50,53-57]. Furthermore, some studies found that there was no association between COX-2 expression and VEGF-C expression or COX-2 *and *VEGF-C mRNA levels in several types of cancer [57-59]. In our study, we did not find correlations between COX-2 and VEGF-C, or COX-2 and LVD. Though COX-2 expression was associated with survival time, COX-2 was not correlated with VEGF-C or LVD. Our data did not show that overexpression of COX-2 promotes tumor lymphangiogenesis through an up-regulation of VEGF-C expression in gastric carcinoma. This difference is based upon the smaller number of specimens examined (mostly *n *< 100), a biased selection of patients, different scoring systems, or different antibodies used. In addition, most studies were retrospective.

## Conclusions

The overexpression of VEGF-C and COX-2 has been found in gastric carcinoma tissues. Age, COX-2 and peritumoral LVD were independent prognostic factors for human gastric carcinoma. Although COX-2 expression was associated with survival time, it was not correlated with VEGF-C or peritumoral LVD. Our data did not show that overexpression of COX-2 promotes tumor lymphangiogenesis through an up-regulation of VEGF-C expression in gastric carcinoma. These findings warrant further larger studies to clarify the association between COX-2 and lymphangiogenesis in gastric cancer.

## Competing interests

The authors declare that they have no competing interests.

## Authors' contributions

HG, XC and MH designed this study and carried out immnunohistochemistry staining, performed the statistical analysis, collected clinical information and drafted the manuscript. JZ, MJ, YY, DC participated in immunohistochemistry staining, the patients follow up and the statistical analysis. All authors read and approved the final manuscript.
